# Resveratrol Prevents Right Ventricle Remodeling and Dysfunction in Monocrotaline-Induced Pulmonary Arterial Hypertension with a Limited Improvement in the Lung Vasculature

**DOI:** 10.1155/2020/1841527

**Published:** 2020-02-03

**Authors:** Eduardo Vázquez-Garza, Judith Bernal-Ramírez, Carlos Jerjes-Sánchez, Omar Lozano, Edgar Acuña-Morín, Mariana Vanoye-Tamez, Martín R. Ramos-González, Héctor Chapoy-Villanueva, Luis Pérez-Plata, Luis Sánchez-Trujillo, Guillermo Torre-Amione, Alicia Ramírez-Rivera, Gerardo García-Rivas

**Affiliations:** ^1^Tecnologico de Monterrey, Escuela de Medicina y Ciencias de la Salud, Ave. Morones Prieto 3000, 64710 Monterrey, N.L., Mexico; ^2^Centro de Investigación Biomédica, Hospital Zambrano-Hellion, Tec Salud, Tecnologico de Monterrey, Batallón San Patricio 112 Col. Real de San Agustín, 66278 San Pedro Garza García, N.L., Mexico; ^3^Unidad de Investigación Clínica en Medicina SC, 64718 Monterrey, N.L., Mexico; ^4^Weill Cornell Medical College, Methodist DeBakey Heart & Vascular Center, The Methodist Hospital, Houston, 77030 TX, USA; ^5^Unidad de Investigación Clínica en Medicina, 64718 Monterrey, N.L., Mexico

## Abstract

Pulmonary arterial hypertension (PAH) is a life-threatening disease that is characterized by an increase in pulmonary vascular pressure, leading to ventricular failure and high morbidity and mortality. Resveratrol, a phenolic compound and a sirtuin 1 pathway activator, has known dietary benefits and is used as a treatment for anti-inflammatory and cardiovascular diseases. Its therapeutic effects have been published in the scientific literature; however, its benefits in PAH are yet to be precisely elucidated. Using a murine model of PAH induced by monocrotaline, the macroscopic and microscopic effects of a daily oral dose of resveratrol in rats with PAH were evaluated by determining its impact on the lungs and the right and left ventricular function. While most literature has focused on smooth muscle cell mechanisms and lung pathology, our results highlight the relevance of therapy-mediated improvement of right ventricle and isolated cardiomyocyte physiology in both ventricles. Although significant differences in the pulmonary architecture were not identified either micro- or macroscopically, the effects of resveratrol on right ventricular function and remodeling were observed to be beneficial. The values for the volume, diameter, and contractility of the right ventricular cardiomyocytes returned to those of the control group, suggesting that resveratrol has a protective effect against ventricular dysfunction and pathological remodeling changes in PAH. The effect of resveratrol in the right ventricle delayed the progression of findings associated with right heart failure and had a limited positive effect on the architecture of the lungs. The use of resveratrol could be considered a future potential adjunct therapy, especially when the challenges to making a diagnosis and the current therapy limitations for PAH are taken into consideration.

## 1. Introduction

Pulmonary arterial hypertension (PAH) is a rare but progressive and often fatal pulmonary vascular disease [1]. PAH is characterized by a progressive increase in pulmonary vascular resistance and pulmonary arterial pressure, with secondary vascular and right ventricular (RV) remodeling, RV dysfunction, heart failure syndromes, and, finally, premature death [2]. Currently, approved therapies target three main pathways important in endothelial function: the prostacyclin and nitric oxide pathways, which are underexpressed, and the endothelin pathway, which is overexpressed in PAH patients [3]. PAH triggers a series of events on RV function, including activation of several signaling pathways that regulate cell growth, metabolism, extracellular matrix remodeling, and energy production [4, 5]. Right heart failure syndrome emerges in the setting of ischemia, alterations in substrate and mitochondrial energy metabolism, increased free oxygen radicals, increased cell loss, downregulation of adrenergic receptors, increased inflammation and fibrosis, and pathologic microRNA expression [4]. Current therapeutic schemes have not been able to regulate these mechanisms in the long term; therefore, there is a need for more successful strategies to manage right ventricular heart failure in the future [4].

Although the current treatment improves quality of life and survival [6, 7], a therapeutic approach to improve the RV function is lacking. Resveratrol (RES) is a phenolic compound with a known cardioprotective effect in several cardiovascular diseases [8]. However, its primary mechanisms of action have yet to be fully elucidated but include sirtuin modulation, reactive oxygen species (ROS) scavenging, and antioxidant mechanisms [9, 10]. The *in vitro* use of RES has been demonstrated to reduce oxidative stress and increase cell survival, inhibiting apoptosis and modulating the cell cycle in several cell lines [11]. RES has also been reported to have antifibrotic and anti-inflammatory effects *in vivo* [12]. This compound has been evaluated in some PAH animal models for its ability to decrease lung damage in the tissue or pulmonary trunk [13], but the molecular mechanism of cardioprotection afforded by RES remains only partially understood. Thus, in this study, the effect of RES in a PAH model on the lungs and ventricles was assessed in its ability to delay PAH progression. To achieve this, we performed an echocardiographic assessment to evaluate ventricular function, macroscopic and histologic changes, as well as contractile modifications, and biomarker expression in isolated cells. RES was demonstrated to be preferentially cardioprotective of the function and structure of the right ventricle, and it was shown to have a limited effect on the pulmonary vasculature.

## 2. Materials and Methods

### 2.1. Murine Model of Pulmonary Hypertension

All animal studies were approved by the Internal Committee for Care and Handling of Laboratory Animals of the School of Medicine of the Tecnologico de Monterrey (Protocol no. 2017-006) and were performed following the Mexican National Laboratory Animal Health Guidelines (NOM 062-ZOO 1999). Experiments were performed on adult male Sprague–Dawley rats (Bioinvert, MX), weighing >300 g. Pulmonary hypertension was induced by a single subcutaneous injection of monocrotaline (MC) (60 mg/kg body weight) diluted in dimethylsulfoxide (DMSO, Sigma-Aldrich, St. Louis, MO, USA). DMSO was used with the same volume for both groups of control rats and only RES rats. Animals were kept in a controlled temperature environment with a 12 h light cycle. Water and food were given ad libitum. To assess the effect of RES (Trans-isomer, RyTLabs), we divided the specimens into four groups: control rats (CTRL, *n* = 12), monocrotaline-treated rats (PAH, *n* = 12), rats treated with MC and RES (20 mg/kg per day, by gavage) (PAH+RES, *n* = 11), and only RES rats (20 mg/kg/day, by gavage) (RES, *n* = 13), from day 1 to day 42 after injection. All animals were observed for general appearance and respiratory symptomatology. Disease progression was characterized by anatomical postmortem data and echocardiography, which correlate strongly with the right heart catheterization measurements.

### 2.2. Echocardiographic Assessment of Cardiac Function

Noninvasive, transthoracic cardiac ultrasonography was performed 35 days after MC/DMSO injection, with a Philips EnVisor Ultrasound (Philips Healthcare, Andover, MA) equipped with a 12 MHz S-type transducer, under 1-3% sevoflurane anesthesia. After placing the animal on a thermal pad, with the chest shaved and using ultrasound transmission gel, standard views recommended by the American Society of Echocardiography were obtained. A parasternal short-axis view at the level of the great vessels was used to measure pulmonary artery flow, using pulse wave Doppler mode with a sample gate of 1.0 mm just proximal to the pulmonary valve. Here, we measured the pulmonary artery velocity time integral (VTI), pulmonary ejection time (ET), peak pulmonary flow velocity, and the pulmonary artery acceleration time (PAAT). A ratio between PAAT and ET was obtained, and the mean pulmonary artery pressure (mPAP) was estimated using the formula mPAP = 58.7 − (1.21 × PAAT). By combining pulmonary artery velocity-time integral, pulmonary artery area, and heart rate, echocardiographically derived cardiac output was determined, as previously published [14]. RV free wall thickness was measured at end-diastole from the parasternal long-axis view using M-mode. The apical four-chamber view was employed to measure the end-diastolic RV diameter and M-mode-derived tricuspid annular plane systolic excursion (TAPSE). Left ventricle (LV) diameters and fractional shortening (FS = diastolic LVID − systolic LVID/diastolic LVID × 100 [LVID (LV internal diameter)]) were measured with M-mode from the short-axis view at the level of the papillary muscles. Subsequently, the echocardiographic RV/LV end-diastolic diameter ratio was calculated and used as an assessment of RV enlargement.

### 2.3. Histological Preparations

After 42 days of RES treatment, the specimens were euthanized. Hearts were quickly excised from the rats after being anesthetized with inhaled 5% sevoflurane and sodium heparin (1000 U/kg). The heart and lungs were dissected and weighed. The RV and LV were identified and isolated for different preparations. The sections for histological findings were fixed in 4% (wt/vol) paraformaldehyde in PBS for at least 2 hours at room temperature, transferred to 70% ethanol, embedded in paraffin, and processed for hematoxylin/eosin (H&E) and Masson's trichrome staining. Fibrotic index assessment was performed following previously published data [15]; in brief, microphotographs were acquired using an Imager Z1 Zeiss microscope with an AxioCam HRm and microphotograph processing with the AxioVision software. To assess fibrosis, we used a semiquantitative approach; after staining with Masson's trichrome, we take microphotographs of the whole slide at 2.5x, and the image is then decomposed in at least seven fields at 5x. After the photos were taken, we quantified the number of blue and red pixels, and the results were recorded to make a ratio of blue%/red% using ImageJ software. Data correspond to the analysis of 2 blinded analysts and three different fields. Cardiomyocyte area was assessed using H&E slides. Microphotography of the papillary muscles was taken at 10x; only cells with a complete visible cytoplasm and central nuclei were considered. At least ten cells per photography were counted at two different levels. All slides were analyzed using an object carrier with a capacity for 7 slides, for their respective batches.

Regarding lung sections, the primary lung architecture was assessed for each group using the H&E-stained slides. Predominant findings included inflammatory infiltration and proliferation of the smooth muscular medial layer of the lung arterioles. We quantified the amount of these arterioles in seven random fields; vessels of an average 100 *μ*m were selected to analyze diameter, luminal area, and occlusion. Occlusion was assessed by at least seven measurements of the medial layer thickness for the average.

### 2.4. Cardiomyocyte Isolation

Ventricular myocytes were isolated following previously described methods [16]. The hearts were excised and mounted on a Langendorff apparatus and perfused with Tyrode medium (TM), in mM: 128 NaCl, 0.4 NaH_2_PO_4_, 6 glucose, 5.4 KCl, 0.5 MgCl-6H_2_O, 5 creatinine, 5 taurine, and 25 HEPES, pH 7.4 at 37°C, for 5 min and digested by 0.1% collagenase type II (Worthington Biochemical, Lakewood, NJ) dissolved in TM. Afterwards, the RV and LV were dissected, and their cells mechanically disaggregated. Cardiomyocytes were rinsed with TM plus 0.1% albumin solution at increasing Ca^2+^ concentrations (0.25, 0.5, and 1 mM). Only rod-shaped cells were used in the studies. All the confocal measurements were acquired using a Leica TCS SP5 confocal microscope equipped with a D-apochromatic 63x, 1.2 NA, oil objective (Leica Microsystems, Wetzlar, Germany). To assess cell volume, freshly isolated cardiomyocytes were incubated in TM with 5 *μ*M calcein-AM (Life Technologies, Carlsbad, CA, USA) at room temperature (RT) for 30 min as previously described [17]. Then, cells were washed with a fluorophore-free and calcium-free solution and images were taken at 400 Hz, obtaining a stack of 2D images of 1 *μ*m section thickness every 1 *μ*m in the *z*-axis, covering the whole cell depth. A 488 nm wavelength was used to excite the fluorophore, and its emission was collected at 500-600 nm. Cell volume was evaluated as previously described [18]. Freshly isolated cardiomyocytes were incubated in TM (1 mM Ca^2+^) with 10 *μ*M Fluo-4 AM (Life Technologies, Carlsbad, CA, USA) for 45 min at RT. Afterwards, the cells were washed with a fluorophore-free solution, plated on laminin-covered glass coverslips and mounted in a superfusion chamber. Excitation and emission wavelengths were 488 nm and 500-600 nm, respectively. Cell shortening was evaluated under field stimulation (MYP100 MyoPacer Field Stimulator; IonOptix, Milton, MA). The cells were evaluated under field stimulation at 0.5, 1, and 2 Hz, and line-scan images were recorded along the longitudinal axis of the cell at 400 Hz with a one *μ*m section thickness. Fluorescence data were normalized as Δ*F*/*F*0, where *F* is fluorescence intensity; all confocal microscopy images were analyzed using ImageJ.

### 2.5. Western Blotting

Total heart protein from right ventricles (30 *μ*g) was resolved on SDS-PAGE gel 15% and transferred onto a PVDF membrane at 300 mA for 2 hours and incubated with anti-Acetylated-Lysine protein antibody (9441S, Cell Signaling) (1 : 2000). The membrane was washed three times for 10 min with PBS-0.5% Tween 20 and subsequently probed with an HPR-conjugated secondary antibody anti-rabbit IgG 1 : 5000 (sc-2004, Santa Cruz) for 2 hours at room temperature. After washing three times for 10 min, the blots were developed with SuperSignal West Dura Extended Duration Substrate (Thermo Fisher Scientific, USA) and quantified by using a BioSpectrum 415 Image Acquisition System (UVP, Upland, CA, USA). Anti-GAPDH antibody (1 : 2000) (ab9484, Abcam) was used as a loading control.

### 2.6. Real-Time Polymerase Chain Reaction (PCR) Analysis

#### 2.6.1. RNA Isolation, Reverse Transcription, and Quantitative PCR (qPCR)

The total RNA from the tissue of the right ventricles was isolated using a TRIzol Reagent (15596026, Invitrogen). The purity of all samples was confirmed measuring their 260/280 nm absorbance ratio using a Take3 multivolume plate in a Synergy HT microplate reader (BioTek Instruments). The cDNA was reverse-transcribed from 1 *μ*g of total RNA using the SensiFAST cDNA Synthesis Kit (BIO-65053, Bioline). The qPCR reaction was performed using the SensiFAST SYBR Lo-ROX Kit (BIO-94020, Bioline) in a QuantStudio 3 RT PCR System (Thermo Fisher Scientific) and the data analyzed by the 2^−*ΔΔ*Ct^ method to estimate each gene's mRNA expression. The primers were synthesized by T4 Oligo (Mexico). All primer sequences for BNP, collagen 1, IL-1*β*, IL-10, troponin C, Sirt1, and HPRT as housekeeping genes are detailed in Supplementary [Supplementary-material supplementary-material-1].

#### 2.6.2. Reagents

All chemical reagents were purchased from Sigma-Aldrich (St. Louis, MO, USA) unless otherwise stated.

### 2.7. Statistical Analysis

Statistical data are presented as the mean ± SEM. Comparisons between means were made by unpaired Student's *t*-test or one-way ANOVA followed by Dunnett's, Tukey's, or Bonferroni's post hoc tests when appropriate to compare experimental groups. Differences were considered significant when *p* < 0.05. Data processing, graphs, and statistical analysis were performed with GraphPad Prism (V.5.01; La Jolla, CA, USA).

## 3. Results

### 3.1. Resveratrol Had a Limited Effect on the Development of a Monocrotaline-Induced PAH Changes in the Vascular Architecture of the Lungs and the Echocardiographic Pulmonary Artery Values

**Figure 1:**
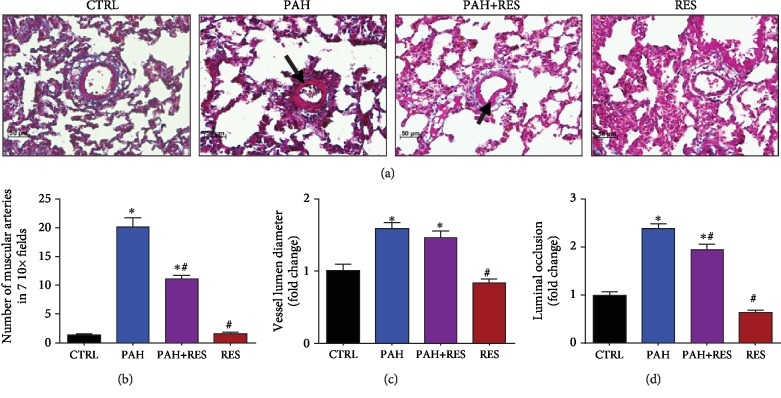
There is a limited effect of RES exerted in the lung vessel histopathology structure. (a) Representative microphotographs of pulmonary blood vessels. PAH induced hypertrophy and proliferation of the tunica media; this effect is decreased by RES. 20x magnification; H&E staining. Arrows indicate the muscularized vessel wall. (b) Amount of muscular arteries in 7 random fields in lung tissue. (c) Diameter of pulmonary blood vessels. (d) Luminal occlusion by the media layer in lung arteries. The values are given as the mean and fold change ± SEM; ^∗^*p* < 0.05 vs. control; ^#^*p* < 0.05 vs. PAH; *n* = 15 for CTRL, PAH, and PAH+RES; *n* = 11 for RES.

The study duration for this PAH model was 42 days as this was an adequate amount of time for phenotypic changes (i.e., cyanosis in the extremities and weight loss) to take place. An increase in the weight of the heart and lungs was identified as a specific macroscopic change. Compared to the untreated control group (CTRL) (1.4 ± 0.2 g), heart weight increased by 21% in the PAH group (1.7 ± 0.2 g) and 35% with 1.7 ± 0.3 g for the PAH+RES group. Lungs weight followed the same trend: a weight increase of 2.0 ± 0.3 g was reported for the CTRL group, 45% increase was observed for the PAH group (2.9 ± 0.3 g), and a 60% increase was seen for the PAH+RES group (3.2 ± 0.4 g). Normalized organ weight and the weight of each specimen showed the same trend (data not shown). There were no statistically significant changes in these values between the PAH+RES and PAH groups and no differences between the CTRL group and the group treated only with RES. To exclude other cardiovascular pathologies, systemic, systolic, and diastolic blood pressure and heart rate values were evaluated. No differences were observed between the groups (data not shown). The pathognomonic findings for lung vasculature in the PAH model included an increase in the muscularized arteries, an increase in the lumen diameter, and a concomitant decrease in luminal occlusion in the media layer. The changes were due to the proliferation of the smooth muscle cells and a slight increase in vessel diameter. Representative microphotographs can be seen in Figure 1(a). RES was unable to avoid the transformation of healthy vessels into muscularized arteries; however, it diminished the amount of them (i.e., an 11.2-fold increase in the PAH+RES group and a 20-fold increase in the PAH group, representing a 56% decrease mediated by RES between these two groups), when compared with its effect on the CTRL group (Figure 1(b)). There was a reduced effect of RES on the vascular lumen diameter (Figure 1(c)). Although there was a noticeable increase of the PAH group compared to the CTRL group, this value was not significantly decreased by RES, having 57.2 ± 2.3 *μ*m (a 1.59-fold increase in the PAH group and a 1.47-fold increase in the PAH+RES group). This represented a 4% decrease in terms of the effect of RES on the PAH phenotype (i.e., 73.5 ± 2.4 *μ*m vs. 70.7 ± 2.6 *μ*m). Luminal occlusion followed a similar trend, with at least a 2.3-fold increase in the PAH group and a 1.9-fold increase in the PAH+RES group compared to CTRL; this represented a 13% decrease in occlusion due to the effect of RES when the PAH+RES and PAH groups were compared (i.e., 30.5 ± 0.9% vs. 26.6 ± 0.9%, respectively) (Figure 1(d)). The rodents treated only with RES showed no differences compared to the CTRL for all the variables.

These data correlate with the echocardiographic values pertaining to the pulmonary artery. Compared with the CTRL group, the PAAT decreased significantly in the PAH and PAH+RES groups (i.e., by 40% and 43%, respectively). The PAAT/ET ratio was seen to reduce in correlation with an increase in pulmonary vascular resistance in both groups, but the change was increased higher in the PAH group than in the PAH+RES group (29% and 25%, respectively). The mPAP markedly increased in the PAH and PAH+RES groups (71% and 43%, respectively) compared to the CTRL group. The mPAP for both groups was estimated to be higher than 20 mmHg, but this change was without statistical significance (Table 1).

### 3.2. Resveratrol Treatment Improves Right Ventricular Remodeling and Function

**Figure 2:**
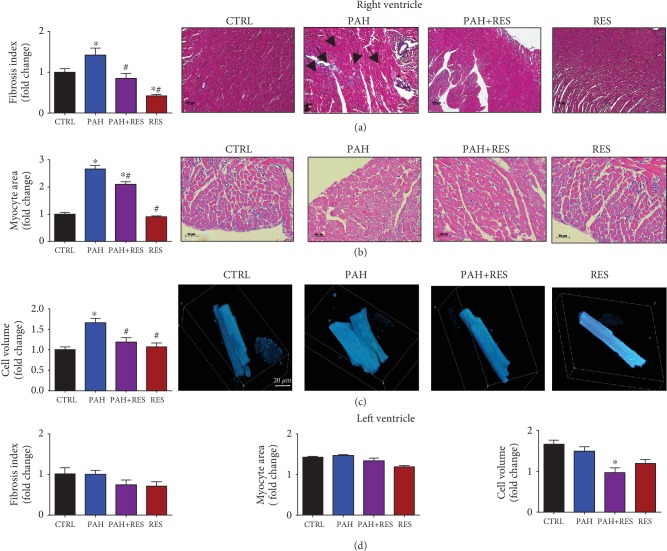
RES reduces the fibrotic index and myocyte hypertrophy in RV of PAH-treated specimens. (a) Fibrotic index. Representative right ventricles stained with Masson's trichrome of all treated groups (5x). Black arrows indicate the zones of fibrotic tissue. (b) Myocyte area. Representative cross-section cardiomyocytes from groups (H&E, 10x). (c) Cell volume analysis. Representative right ventricle cardiomyocytes stained with calcein and analyzed by confocal microscopy. (d) Unchanged LV morphological features in the PAH model and the lack of effect of RES on these features. Fibrotic index, myocyte area, and isolated cell volume. All data have been normalized to RV CTRL mean values. The values are given as the mean and fold change ± SEM (*n* = 15 for CTRL, PAH, and PAH+RES; n = 11 for RES). The values are given as the mean and fold change ± SEM; ^∗^*p* < 0.05 vs. CTRL; ^#^*p* < 0.05 vs PAH.

The values found using TAPSE, a surrogate measurement of right ventricular performance, worsened significantly by 31% in the PAH group compared to the CTRL group. In contrast with PA hemodynamics, this value improved considerably by 46% in the PAH+RES group, compared to PAH. RV free wall thickness increased markedly by 60% in the PAH group compared with the CTRL group. However, in the treated PAH+RES group, RV free wall thickness decreased significantly by 25% compared with the PAH group. The end-diastolic diameter of the RV and the ratio of the end-diastolic diameter for the RV/LV increased significantly in the PAH group compared to the CTRL group. A significant difference was not observed for both parameters in the PAH+RES group compared to the CTRL group (Table 2). The effect of RES, at the microscopic level, on the tissues and isolated cells is depicted in Figure 2(a). To assess tissue remodeling, the ventricular wall sections were stained with Masson's trichrome. A multiple-fold increase in fibrosis was observed in the RV in PAH. RES treatment kept the fibrosis at CTRL levels in the PAH+RES phenotype, while the effect of RES treatment alone was the same as that of the CTRL group. The LV in all groups remained unchanged (Figure 2(d)).

### 3.3. Resveratrol Restored Right Ventricular Cardiomyocyte Structure and Contractile Function in Rodents with Pulmonary Arterial Hypertension

**Figure 3:**
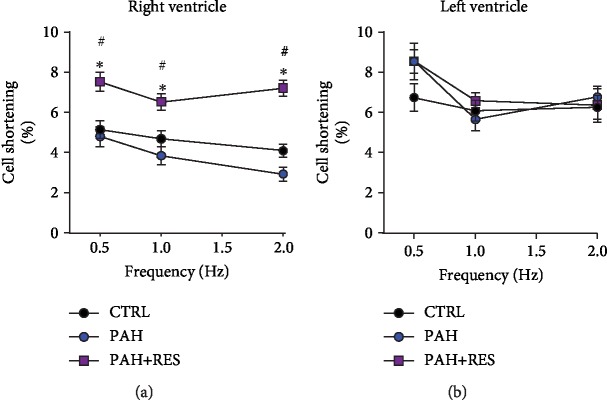
RES improves cardiomyocyte shortening isolated from RV, and had with on LV isolated cardiomyocytes. Percentage of cell shortening after 1 Hz stimulation in isolated cardiomyocyte from the (a) right ventricle and (b) left ventricle. The values are given as the mean ± SEM (^∗^*p* < 0.05 vs. control, #*p* < 0.05 vs. PAH; *n* = 26-44 cells from 2 animals for CTRL, *n* = 15-26 cells from 2-3 animals for PAH, and *n* = 19-61 cells from 2-4 animals for PAH+RES).

The cardiomyocyte area was analyzed using H&E-stained slides at the level of the papillary muscles. The PAH group had this parameter with at least a two-fold increase (600 ± 23 *μ*m^2^) compared to the CTRL group (290 ± 11 *μ*m^2^). The cardiomyocyte area decreased by 17% in the PAH + RES group (498 ± 17.8 *μ*m^2^) compared to the PAH group (Figure 2(b)). In contrast, a difference between any of the groups was not observed in the LV myocyte area (Figure 2(d)). The cellular volume of the isolated RV cardiomyocytes in rodents with PAH assessed using confocal microscopy reflected a 1.7-fold increase compared to the CTRL group (34.8 ± 1.9 vs. 23.6 ± 1.1 fL, respectively). The PAH+RES group was seen to have a 13% decrease in volume compared to the PAH phenotype (26.8 ± 1.7 fL), and there was no statistical difference with the CTRL group (Figure 2(c)). There were no differences observed between the LV groups (Figure 2(d)). These changes could indicate the prevention or delay of tissue remodeling exerted by RES. Taken together, these data prompted the analysis of functional cell shortening in the isolated cells. The effect of increased stimulation frequency on cell contractility in the RV and LV cells was evaluated. The cells were paced at 0.5, 1.0, and 2.0 Hz. The RV CTRL cells had respective values of 5.1 ± 2.4%, 4.6 ± 2.4%, and 4.1 ± 1.7%. There were no changes in the RES-only group compared to the CTRL group (data not shown). In our animal model, the PAH group exhibited a contractility decrease of 8% at 0.5 Hz, 18% at 1.0 Hz, and 39% at 2.0 Hz compared to the CTRL group. Cell shortening in all paces was demonstrated to be significantly improved in the PAH+RES group compared to the other groups. Compared to PAH in this regard, there was a respective increase of 59% for 0.5 Hz, 71% for 1.0 Hz, and 148% for 2.0 Hz in the PAH+RES phenotype (Figure 3(a)). A statistically significant difference was not seen regarding the effect of PAH or RES on the LV cells (Figure 3(b)).

### 3.4. Inflammatory and Remodeling Effect of RES in RV Tissue from Specimens with PAH

**Figure 4:**
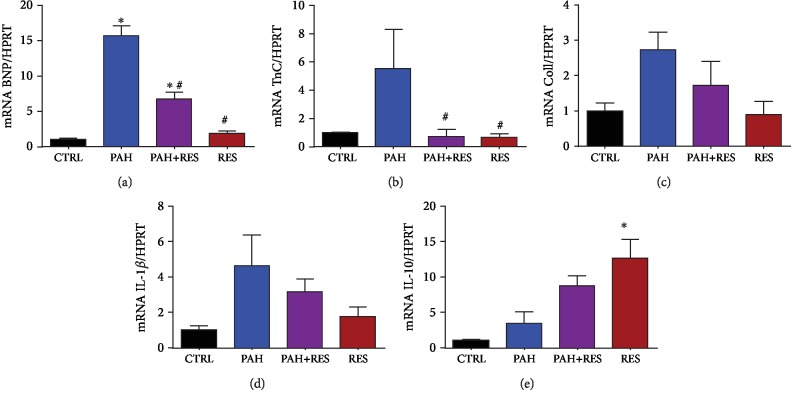
RES modulates the decrease of tissue remodeling and inflammatory mRNA on the RV of PAH-treated specimens. qPCR analysis of RV from the tissue samples show (a) BNP, (b) troponin C, (c) collagen type 1, (d) IL-1*β*, and (e) IL-10. All data have been normalized to RV CTRL mean values. The values are given as the mean and fold change ± SEM (*n* = 4 for CTRL, *n* = 3 for PAH and PAH+RES, and *n* = 6 for RES). The values are given as the mean and fold change ± SEM; ^∗^*p* < 0.05 vs. control; ^#^*p* < 0.05 vs. PAH.

We performed qPCR from RV tissue to analyze the remodeling and inflammatory markers. We selected BNP, troponin C1 (Tnnc1), and collagen 1 to analyze the hypertrophy mediated by MC. For the inflammatory markers, we chose pro-inflammatory IL-1*β* and anti-inflammatory IL-10. There was a significant RES-mediated decrease in the PAH+RES group of the following mRNAs compared to PAH phenotype: BNP (6.7 ± 1.02 vs. 15.7 ± 1.5), Tnnc1 (0.7 ± 0.5 vs. 5.5 ± 2.7), collagen 1 (1.7 ± 0.7 vs. 2.7 ± 0.5), IL-1*β* (3.2 ± 0.7 vs. 4.6 ± 1.7), and an increase in IL-10 (8.7 ± 1.5 vs. 3.5 ± 1.6) (Figures 4(a)–4(e)).

### 3.5. There Is a RES-Mediated Increase in SIRT1 in Treated PAH RV

**Figure 5:**
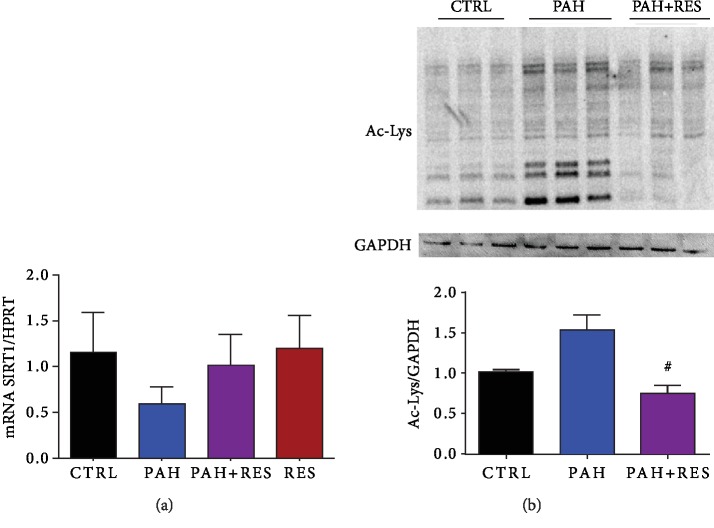
There is a decrease in the mediated SIRT1 deacetylation. (a) qPCR analysis of SIRT1 mRNA expression on heart tissue. (b) Representative western blot membrane of Ac lysine of heart tissue proteins and below the acetylation profile in heart tissue in fold change. For (a), *n* = 4 for all groups; for (a), *n* = 3 for all groups. The values are given as the mean and fold change ± SEM; ^∗^*p* < 0.05 vs. control; ^#^*p* < 0.05 vs PAH.

We found an expected downregulation of SIRT1 mRNA in the RV of PAH phenotypic rats compared to CTRL. This decrease (0.5 ± 0.2) was abrogated by the treatment of RES in these specimens (0.8 ± 0.3) (Figure 5(a)). To evaluate the effect of the decrease in SIRT1 expression, we performed a western blot of the acetylation profile. Treatment of PAH with RES prevented the increased acetylation of the RV, remaining at the level of the CTRL group (Figure 5(b)).

## 4. Discussion and Conclusion

The use of animal models, like the MC-induced PAH in rats has been an alternative to characterize and explore the physiopathology and therapeutics against PAH [19]. The majority of the literature has focused on relevant topics such as pulmonary changes in the smooth muscle of the vessels and the incidence of fibrosis [13, 20, 21]. However, it is important to highlight the lack of protocols focused on improving RV function, a critical parameter for outcome and survival [22]. An example of the clinical relevance of improving the ventricular function comes from the reversible effects after treating chronic thromboembolic pulmonary hypertension (CT-PH), where careful removal of the thrombus reverses the compromised ventricular function and its remodeling. Therefore, there is a need to continue the development of coadjuvant treatments for RHF and improve their mechanisms of delivery [10, 22, 23].

A decision was made to address these issues by studying the underlying mechanisms associated with RES treatment on the cardiovascular and pulmonary system in a valid model of PAH. The use of RES as an adjuvant in a therapeutic setting owing to its multiple effects as a ROS scavenger, mitochondrial agent, and cell cycle modulator is ongoing and constitutes promising research [10]. Varying doses of RES [14, 24, 25] and administration routes [25–27] have been used for numerous therapeutic purposes and diseases. Several RES doses for PAH models have been described in the literature, and variable results have been reported [28]. The cause of this variation relates to the MC dosage, the initial weight of the specimen, and the duration of the study.

For the model in this study, RES at a dose of 20 mg/kg/day by gavage was used, a dose that has been used elsewhere [29]. According to the findings of the current study, heart and lung weight increased in both the PAH and PAH+RES groups. The effect of RES was not significant, and this phenomenon has been reported in other research [13] and may be associated with several factors. An explanation for this is that a higher RES dosage is required for a macroscopic impact.

Consistent and pathognomonic results were found for the vascular bed of the lungs for all groups treated with the PAH phenotype [30, 31]. RES had a minimal effect in reversing them, other authors found that RES had a limited antiremodeling effect on the lungs, and this effect was limited to the medial layer of the pulmonary trunk and did not impact the heart wall structure [13]. In the current study, RES had a partial effect on lung histopathology in the PAH+RES-treated group. Using a higher dose and improving the administration route (i.e., using nebulization therapy) could be an effective way of improving these results.

The finding in the current study that RES had a limited effect on the lungs correlates with ultrasonographic evidence of PA. Echocardiography was chosen as it is a noninvasive tool that can be used to assess RV function. The current study demonstrated that it was a feasible technique that could be used in rats for a PAH evaluation, and this has also been demonstrated by other groups [32]. The PAAT and PAAT/ET ratio are PA hemodynamic parameters that are highly susceptible to changes in pulmonary vascular resistance and impedance [33], and it shortens in correlation with an increase in systolic PAP and mPAP [34]. Although RES prevented the development of specific pathognomonic PAH characteristics, it was insufficient to elicit a significant change in surrogate markers of increased mPAP. Chronic RV pressure overload has been shown to lead to a gradual change in RV phenotype, which ultimately resulted in RV-arterial uncoupling and subsequent functional deterioration [35]. A significantly low value of mPAP was found in the PAH group in the current research, and more importantly, an improvement of this parameter was reported for the PAH+RES group, suggesting that treatment with RES prevented systolic failure, commonly observed in the advanced stages of PAH. Interestingly, this value was even higher in the RES-only treatment group, which could indicate that this polyphenol not only prevents failure but is also a potential enhancer of RV function [35]. RV free wall thickness, an objective reflection of RV hypertrophy and remodeling [34], was markedly increased in rodents with PAH, which is consistent with remodeling secondary to pressure overload. Most importantly, and in contrast with the findings of the PAH group, RES treatment attenuated RV hypertrophy induced by high mPAP, and a significant difference with the CTRL group was not observed. This can be secondary to fewer fibrotic changes, which is consistent with previous *in vitro* research on the impact of RES on cardiac fibrosis [36]. An increase in the RV end-diastolic diameter was observed in the PAH model, and this increase was significant when compared to the untreated controls, reaching a ratio of ≥1, which is associated with an increased risk of adverse clinical events, while being a marker for poor prognosis [37]. Dilation and the increased ratio were not present in the PAH+RES group. Therefore, even with the increased mPAP identified in the PAH+RES group, the RV was able to endure it and prevent pathologic remodeling. A further study in this direction is to investigate the effect of RES on cardiac strain because it is possible that enhancement of these variables by RES could explain the increased capacity of RV to manage elevated pulmonary pressure. The LV echocardiography findings did not show any change associated with increased mPAP with RES treatment. Even though its diameter can decrease as a consequence of RV dilatation or the presence of pericardial effusion, this effect in the PAH groups was not seen upon analysis. One explanation for the absence of these findings and for negative ventricular interaction is that the model did not allow the development of PAH that was severe enough to cause pericardial effusion or result in higher values of mPAP. More research with a focus on echocardiographic changes is warranted to clarify the changes elicited by MC PAH in LV and LHF.

Signs of increased tissue remodeling consistent with fibrotic changes were identified in the current research as previously reported [38, 39]. Interestingly, these changes focused on the right ventricles of MC-treated rats, while the RES treatment led to a tissue structure that was almost identical to that of the untreated controls. Right heart chambers differ from those on the left side, even in terms of their embryology [40] and primary functions, both physiologically and hemodynamically [41].

RES decreased, or, at the very least, inhibited, the progression of fibrosis in the right ventricles of the PAH+RES group. This finding suggests that pressure on the heart decreased after treatment with RES, and this was the primary reason for validating it using an ultrasonographic assessment in the current research. RES-mediated molecular mechanisms involving TGF-*β* modulation [42, 43] and its effect on the medial vascular layer have been associated with afterload pressure changes [44]. Other pathways have been described, such as a MC-induced upregulation of SphK1-mediated NF-*κ*B activation, albeit not in the scope of our current study [45]. Accompanied by a decrease in tissue remodeling, changes in the myocyte area and isolated cell volume were found with RES treatment. Consistent with the lack of fibrotic changes in the LV, these effects were also absent in these chambers. The results showed that individual cell volume and the area in the left ventricular cells in all the studied groups increased in comparison with those in the healthy CTRL RV cells. Hypertrophic LV compensation has been reported in heart failure when the MC model was used and is associated with an increase in neurohumoral activation [46]. An interesting finding was that there were no differences between the LV groups (i.e., the PAH and PAH+RES groups). It could be speculated that remodeling changes in the LV could appear later. However, this study duration was twice as long as the typical duration of 21 days. Therefore, this is unlikely. Generally, PAH is characterized by an increase in pulmonary vascular resistance, which causes RV remodeling and leads to RHF. In the current PAH model, compromised RV function, myocyte hypertrophy, and isolated RV cardiomyocytes were identified. Changes in cellular contractility in the LV were not seen. Interestingly, while a decrease in RV fractional shortening by echocardiography [47] is well known, some studies reported an increase in cellular shortening after treatment with MC [48, 49], but this higher contraction force was not sustained at high-stimulation frequencies [48]; this was also observed in the current study (Figure 3). Consistent with these changes in hypertrophy in an inflammatory model like MC, we found increased levels of mRNA of the remodeling markers BNP, collagen 1, Tnn1c, the inflammatory IL-1*β*, and the anti-inflammatory cytokine IL-10. These markers have been reported previously for PAH [50, 51], while the cytokine IL-10 has been linked with the increase of fibrosis and TGF-*β* [52]. RES treatment in PAH decreased the inflammatory markers and modulated a decrease of the remodeling effect (Figure 4). These results also correlate with the effect of RES-mediated SIRT1 upregulation, with a consequent decrease in the acetylation profile (Figure 5(b)). This has been linked to mitochondrial dysfunction concomitant with ventricular dysfunction and heart failure [53].

The increase in myocardial stiffness, as a result of the overexpression of titin, has been proposed as a possible explanation for contractile dysfunction [54, 55] since this large protein is responsible for the passive elasticity of the muscle. Additionally, some changes in calcium handling could be involved. For instance, in the PAH model, faster calcium transients concomitant with increased sarcoplasmic reticulum calcium content and phosphorylation of phospholamban were demonstrated. Moreover, calcium spark frequency was higher in the RV cardiomyocytes from rodents in the PAH group. In this regard, RES has been shown to upregulate the ratio of phosphorylated phospholamban and is accompanied by a significant improved sarcoplasmic reticulum calcium load. In the current study, the RES-treated group showed an increase in cellular shortening; this may have been due to an increase in myocyte stiffness as the RV energetic calcium handling might have been altered. Impaired mitochondrial function due to hypoxia in hypertrophy remodeling has been reported in PAH, which compromises the supply of energy to the tissues [56]. Furthermore, creatine kinase activity and expression have been demonstrated to decrease after MC treatment [57]. This downregulation has been linked to a compromise in ATP/ADP transport between the mitochondria and the myofilaments. RES, besides being a potent antioxidant, is known for its cardioprotective action as it preserves the mitochondrial function by regulating the activity of antioxidant enzymes by reducing ROS [58].

**Figure 6:**
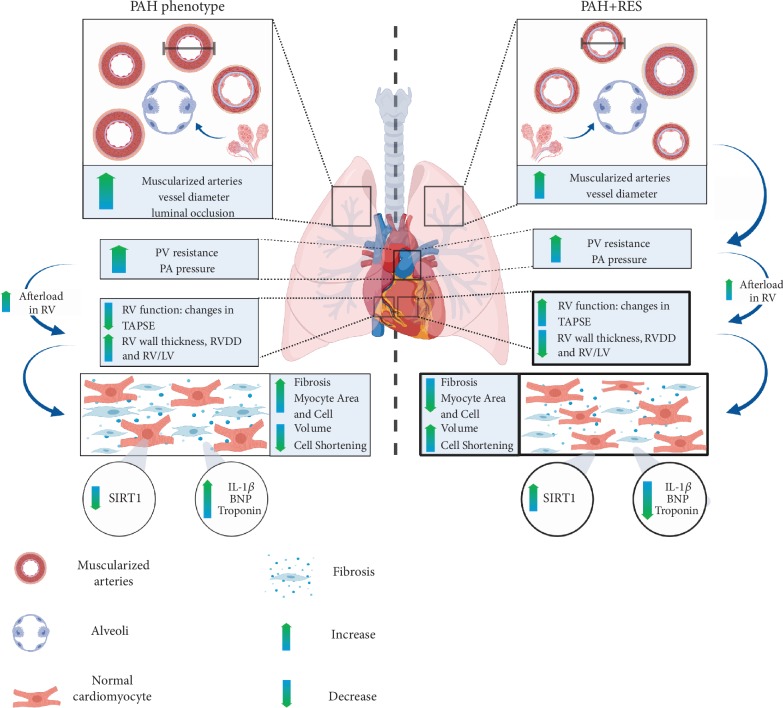
Effects of resveratrol in the PAH phenotype. Monocrotaline-induced PAH is a disease characterized by a progressive remodeling of the pulmonary vasculature, as a consequence of excessive proliferation and migration of pulmonary artery endothelial and smooth muscle cells. With the progression of the disease, the increase of the mean pulmonary artery pressure leads to a chamber pressure overload in the right ventricle (RV). When the optimal RV-arterial coupling is lost, the RV systolic function cannot remain matched to the afterload, and subsequently, dilation of the RV occurs, as well as diastolic dysfunction, secondary to myocardial fibrosis and sarcomeric stiffening. These changes ultimately lead to right heart failure and death. Even though the administration of resveratrol decreased the pathological remodeling of the pulmonary vasculature, it did not change the afterload for the RV (represented in the figure as a change in arrows' thickness). Nevertheless, resveratrol was able to protect directly the RV, improving its function, evidentiated with microscopic changes: less fibrosis, decreased cardiomyocyte area and volume and better cell function, with increased cell shortening, increasing SIRT1-mediated deacetylation, and decreasing inflammatory and remodeling markers. The arrows in the PAH model indicate the changes compared to the CTRL group; the arrows in the PAH+RES model indicate the changes compared to the PAH group.

PAH remains a challenging disease owing to its high morbidity, mortality, and time to diagnose. Although current therapies are improving the quality of life and survival, there is a need to identify adjunctive treatments focussing on RV function. As a result, PAH continues to be revisited, and an increasing number of therapeutic approaches are evaluated annually. However, the current guidelines for pharmacotherapy primarily focus on the vascular effects [59]. Since the disease is usually diagnosed late, a large amount of structural damage in the RV has already taken place. Contrary to some models where RES has been reported to have an effect on the pulmonary trunk [13] and although using an inflammatory model such as MC, the findings of the current study demonstrated that RES improved the RV and had a limited positive effect on the lungs (Figure 6). This finding is crucial since RV function correlates with symptomatology and the prognostic survival of the patients. Focusing on comparative ventricular assessment and isolated cell function, the current study showed how RES-mediated mechanisms might be involved using this model. Some other mechanisms include activation of specific sirtuin pathways like SIRT3, the modulation of cardiac energetics [53], and transcription factors relating to proliferation and the cell cycle [11]. Possible novel targets that focus on RV HF could become an exciting future scope for therapy. The multitargeted nature of RES, as an example of these polyphenolic compounds, holds future potential for novel approaches to this disease [60]. Ongoing research is needed to help characterize the molecular mechanisms and cell bioenergetics of these compounds in PAH and other cardiovascular conditions.

## Figures and Tables

**Table 1:** Echocardiographic measurements of right ventricle outflow tract flow profiles.

	CTRL (*n* = 15)	PAH (*n* = 13)	PAH+RES (*n* = 13)	RES (*n* = 13)
Heart rate (bpm)	315 ± 10	328 ± 18	328 ± 7	318 ± 6.4
Pulmonary artery acceleration time (ms)	35 ± 2.2	21±1.8^∗∗^	20 ± 1.3^∗^	31 ± 2.1^#^
Ejection time (ms)	98.2 ± 5.1	82.9 ± 4.6	80.6 ± 4.6	87.5 ± 5.8
PAAT/ET ratio	0.35 ± 0.02	0.25 ± 0.02^∗^	0.26 ± 0.02	0.35 ± 0.01^#^
Peak gradient (mmHg)	3.1 ± 0.3	3.2 ± 0.2	3.4 ± 0.4	3.5 ± 0.2
Estimated mPAP (mmHg)	21 ± 2	36 ± 2.2^∗^	30 ± 1.1^∗^	21 ± 1.7^#^

PAAT: pulmonary artery acceleration time; ET: ejection time. All data are presented as the mean ± SEM. ^∗^*p* < 0.05 vs. CTRL; ^#^*p* < 0.05 vs. PAH.

**Table 2:** Echocardiographic measurements of right ventricle function.

	CTRL (*n* = 15)	PAH (*n* = 13)	PAH+RES (*n* = 13)	RES (*n* = 13)
Right ventricle outflow tract (mm)	2.9 ± 0.09	3.6 ± 0.2^∗^	3.3 ± 0.08^∗^	3.4 ± 0.08^∗^
Right ventricle output (L/min)	0.19 ± 0.02	0.16 ± 0.02	0.18 ± 0.01	0.24 ± 0.02
TAPSE (mm)	1.9 ± 0.1	1.3 ± 0.1^∗^	1.9 ± 0.1^#^	2.4±0.1^∗,#^
RV wall thickness (mm)	1 ± 0.08	1.6 ± 0.12^∗^	1.2 ± 0.11^#^	1.1 ± 0.09^#^
RV diastolic diameter (mm)	3.4 ± 0.1	4.7 ± 0.2^∗^	3.5 ± 0.2^#^	3.6 ± 0.2^#^
RV systolic diameter (mm)	2.5 ± 0.2	3.1 ± 0.3	2.8 ± 0.3	2.5 ± 0.1
LV posterior wall thickness (mm)	1.7 ± 0.08	1.8 ± 0.1	1.7 ± 0.09	1.6 ± 0.1
LV diastolic diameter (mm)	5.8 ± 0.4	4.6 ± 0.1	5.5 ± 0.3	6.7 ± 0.3^#^
LV systolic diameter (mm)	4 ± 0.6	2.7 ± 0.2	3 ± 0.2	3 ± 0.1
RV/LV diastolic diameter	0.57 ± 0.07	1.04 ± 0.06^∗^	0.65 ± 0.05^#^	0.53 ± 0.02^#^

TAPSE: tricuspid annular plane systolic excursion; RV: right ventricle; LV: left ventricle. All data are presented as the mean ± SEM. ^∗^*p* < 0.05 vs. CTRL; ^#^*p* < 0.05 vs. PAH.

## Data Availability

The data used to support the findings of this study are available from the corresponding author upon request.

## Supplementary Materials

Supplementary Materials Supplementary Table 1: primer sequences for real-time qPCR in heart tissue.
