# Supplementary material for: Resveratrol Prevents Right Ventricle Remodeling and Dysfunction in Monocrotaline-Induced Pulmonary Arterial Hypertension with a Limited Improvement in the Lung Vasculature
Source: Oxid Med Cell Longev. 2020 Feb 3;2020:1841527. doi: 10.1155/2020/1841527 (PMC7023844; doi:10.1155/2020/1841527)
Supplement: Supplementary Materials — Supplementary Table 1: primer sequences for real-time qPCR in heart tissue. [file 1841527.f1.docx]

**SUPPLEMENTARY INFORMATION**

**Supplementary Table 1: Primer sequences for real time q PCR in heart tissue**

**Gene name Forward primer 5´-3´ Reverse primer 5´-3 __________________________________________________________________________________________________**

**HPRT** CGTGATTAGTGATGATGAACC GAGCAAGTCTTTCAGTCCT

**BNP** CTCCAGAACAATCCACGAT CTTGAACTATGTGCCATCTTG

**Collagen** 1 GACTGTCCCAACCCCCAAAA CTTGGGTCCCTCGACTCCTA

**IL1β** AAGCCAACAAGTGGTATTCTCCATG GATCCACACTCTCCAGCTGCA

**IL10** GGTTGCCAAGCCTTGTCAGA ACCTGCTCCACTGCCTTGCT

**Troponin** C GAGCTGTCGGATCTCTTCCG CGATTCGGCCATCGTTGTTC

**Sirt1** GAACCTCTGCCTCATCTA TACTCGCCACCTAACCTA

_______________________________________________________________
